# Gauging the Changing Landscape: Telehealth Perceptions among Hispanic Females with Breast Cancer

**DOI:** 10.3390/ijerph20031713

**Published:** 2023-01-17

**Authors:** France Nguyen-Grozavu, Eunjeong Ko, Andrea Valadez Galindo

**Affiliations:** School of Social Work, San Diego State University, San Diego, CA 92182, USA

**Keywords:** telehealth, challenges/disadvantages, advantages, cancer, COVID-19, hispanic

## Abstract

During the COVID-19 pandemic, telehealth use rapidly grew while its uptake steadily increased in cancer care. Prior research has reported existing racial and ethnic disparities in telehealth, with Hispanics reporting lower rates of use compared to other major racial/ethnic groups. Our study examined the perceived benefits and challenges/disadvantages faced by Hispanic females diagnosed with breast cancer in San Diego County, California. In-depth interviews were conducted with 27 participants, who were mostly Spanish speaking. The recordings of the interviews were transcribed and translated from Spanish to English. Reflexive thematic analyses revealed both potential benefits and challenges of telehealth. The perceived benefits included logistic and financial aspects (such as convenience and time/financial savings), faster access and longer duration spent with clinicians, the availability of family members, and the minimization of COVID-19 risk. The reported challenges/disadvantages of telehealth and the suggested strategies to address them focused on limitations in clinical care, diminished engagement with clinicians, difficulty accessing interpreter services, and technological access and challenges. The COVID-19 pandemic has greatly affected the landscape of how care is provided, with a greater shift to telehealth services. More research is needed to further examine the challenges of telehealth, particularly for groups that are disproportionately affected, to avoid the disruption of patients’ cancer care and to promote a better patient healthcare experience.

## 1. Introduction

COVID-19 has had a major impact on how care is provided, with a shift to a greater dependence on telehealth services. Since the outbreak of the COVID-19 pandemic, there have been over 97 million confirmed cases of COVID-19 infection and over 1.06 million deaths in the United States (US) alone [1]. Hispanics are the second largest racial/ethnic group in the US, making up 18.9% of the total US population [2]. During the initial surge of the COVID-19 pandemic in 2020, the death toll among Hispanics was five times greater than in non-Hispanic Whites [3].

Due to the highly contagious nature of the virus and the ease of transmissibility of this airborne disease [4], outpatient healthcare services transitioned to more telehealth appointments and restricted in-person visits. It was reported that health centers funded by the Health Resources and Services Administration experienced a 154% increase in telehealth visits during the first quarter of 2020 compared to the prior year [5]. However, the adoption of telehealth has been slower in the field of cancer compared to other fields, but the uptake of telehealth has been steadily increasing in cancer care [6]. The array of cancer care offered via telehealth ranges from remote chemotherapy supervision to symptom and disease management to survivorship and palliative care [7]. Cancer patients reported overall satisfaction with telehealth [8,9] and supported the use of telehealth in the future [9,10].

The reported advantages of telehealth were multifold, such as time, cost, and travel savings [9,11,12]. In addition, telehealth was perceived as beneficial by taking place in a comfortable home environment [9,11] as well as avoidance of potential exposure to the COVID-19 virus [9,11,13,14]. Despite the benefits of telehealth, patients with cancer face challenges in adapting to the changing healthcare environment during the pandemic. Cancer patients’ concerns and challenges relating to telehealth include limitations in conducting physical examinations [9,15,16,17], building a connection in the patient–clinician relationship [9,11,18], providing a hard copy of the prescription for medication [9], and having poor internet connectivity [9].

A growing body of literature has found that telehealth use via video is challenging for certain populations, such as older adults [19], those with less than a high school education or limited English proficiency, and among racial and ethnic minorities due to digital literacy, accessibility, and the operation of technology [20,21,22,23]. Compared to Whites, racial and ethnic disparities in accessing the internet and technology exist, where Black and Hispanic Americans are less likely to own a desktop or laptop computer or have a broadband connection at home [24]. A previous study found that, despite positive attitudes toward the use of telehealth, the majority of the Hispanic population in the US and along the US-Mexico border were unfamiliar with the term telehealth [25].

In addition, most telehealth systems have operational limitations for medical interpreters, causing a challenge for patients with limited English proficiency [26]. Patients with limited English proficiency, including Hispanics, were less likely to use telehealth and experienced an overall decrease in quality of care compared to Hispanics who spoke English [14,26,27,28]. Those with limited English proficiency were associated with lower levels of education, higher levels of poverty, and being uninsured [22].

While previous studies provided valuable information about racial and ethnic gaps in access and the use of telehealth [14,26,28], studies with Hispanic cancer patients who may be socioeconomically disadvantaged are limited. With the rapid adoption and sustained uptake of telehealth in oncology, telehealth services continue to be integrated into the standard care practice beyond the COVID-19 pandemic [29]. Hence, it is critical to explore the areas of benefits and challenges related to telehealth use from the perspectives of underserved Hispanic breast cancer patients. This study aims to build upon the existing scientific literature by examining these potential benefits and challenges and highlight proposed strategies to address these challenges, thereby improving the patient healthcare experience with the advent of increasing telehealth use.

## 2. Materials and Methods

### 2.1. Study Design and Setting

A qualitative study based on in-depth interviews was conducted in collaboration with a local nonprofit cancer organization located near the US–Mexico border in San Diego County, California. This organization mainly serves cancer patients with low-socioeconomic status by providing health-related and psychosocial support.

### 2.2. Participants and Recruitment Procedures

Convenience and purposive sampling methods were employed to recruit potential participants. The eligibility criteria included self-identification as a Hispanic female, at least 18 years of age, the receipt of a breast cancer diagnosis, and a display of cognitive competence. A staff member from a partner organization assisted with accessing and recruiting potential participants, and interested persons were provided an IRB-stamped recruitment flyer. Initially, there were 53 individuals who expressed interest in the study and were referred to a bicultural and bilingual research assistant for further screening. Five individuals did not meet the eligibility criteria. Among the remaining 48 potential participants, 8 individuals later changed their minds due to various reasons (i.e., feeling ill or not comfortable), 8 failed to show up for their scheduled interviews, and 5 could not be reached; thus, the total study sample size was 27. The study procedures were approved by the institutional review board at San Diego State University (HS-2021-0049).

### 2.3. Data Collection

In-depth interviews were conducted between March and June 2021. The “stay at-home order” was implemented starting in March 2020 in California, USA, and travel restrictions were put into place, including the US–Mexico border closure during the study period. Interviews were conducted via videoconference (henceforth “video chat”, as referred to by the study participants) or phone call by a trained research assistant using a semi-structured interview guide with probes. The questions included: (1) how has your experience been of having to interact with your healthcare team via telehealth for your cancer care; (probe) (1a) what has been particularly helpful or challenging about communicating with and receiving care via telehealth; (2) how different is your feeling between receiving telehealth and a face-to-face meeting; (3) what would be needed to make telehealth work effectively for you; and (probe) (3a) what types of help might be needed for communication and cancer care via telehealth?

Interviews were conducted either in English or Spanish based on participants’ preference and lasted an average of approximately 40 min to one hour. All interviews were audio-recorded for later transcription. Quality checks were conducted by the principal investigator in consultation with the research assistant to address any questions. Supplementary field notes were documented by the research assistant, such as description about the setting, situational events that occurred during the interview, and participants’ nonverbal behaviors. These notes added contextual information and provided insights for improving and maintaining consistency for future scheduled interviews.

### 2.4. Data Analysis

Each interview was audio-recorded, transcribed, and translated from Spanish to English by another research assistant (different from the interviewer). In order to ensure the accuracy of the translation, the two research assistants engaged in discussion and validation of the translation of the document when deemed unclear. Researchers (FN and EK) employed a reflexive thematic analysis approach to identify emergent themes and salient patterns from the interview transcripts [30]. FN and EK were researchers with prior experience conducting and analyzing transcripts in qualitative research studies. The use of two coders helped establish intercoder reliability.

The manual coding of the data was conducted separately by both coders, and open coding was performed independently by these two coders. Each coder identified and developed an initial list of open codes and corresponding quotes from the transcripts illustrating those codes. The coders continually met to discuss and create a final list of agreed-upon open codes. Both coders also formed their own thematic categories based on their open codes, and eventually a final set of major themes was generated after the two coders reached a consensus. This process, which included combining themes that were similar and rephrasing the themes, improved the clarity and trustworthiness of data interpretation. Lastly, the selected quotes in the Results Section were based on an agreement between the coders as to which quotes best illuminate the final set of themes.

The initial analysis focused on topics in the interview guide and other concepts brought up during the interviews. Topics included (1) the advantages of telehealth use; (2) the challenges/disadvantages of telehealth use; and (3) the suggested improvements to make telehealth more effective. Perceived challenges and potential suggestions to improve telehealth were simultaneously derived. Then, the suggestions proposed were aligned and linked to a reported challenge as a possible strategy to improve telehealth.

## 3. Results

### 3.1. Participant Sociodemographic Characteristics and Cancer-Related Information

The participants’ sociodemographic characteristics and cancer-related information are reported in Table 1. The average age of the participants was 54.4 years old. Almost half of the participants (44.4%; n = 12) completed high school, followed by some college (22%, n = 6). About 59.3% (n = 16) reported an annual household income of USD 30,000 or less. The majority of the in-depth interviews (89%; n = 24) were conducted in Spanish. Two-thirds (66.7%; n = 18) possessed a smartphone, while 22.2% (n = 6) had access to both a smartphone and landline phone, and 11.1% (n = 3) only had a landline phone.

More than half of the sample (55.6%; n = 15) were diagnosed with breast cancer less than one year before their scheduled interview date, and one-fourth (25.9%; n = 7) were diagnosed between one and two years ago. About 41% (n = 11) were diagnosed with stage 2 breast cancer, and about 22% (n = 6) were unsure about their breast cancer stage.

### 3.2. Advantages of Telehealth Use

A summary of the perceived advantages, disadvantages, and suggestions to address the disadvantages is reported in Table 2.

Telehealth services were perceived as beneficial in various ways that are categorized into three main areas: (1) logistical and financial aspects (such as convenience, avoidance of transportation barriers, cost-savings), (2) availability of others (e.g., clinicians, presence of family members at the appointments), and (3) personal safety (in reference to minimizing potential exposure to COVID-19 virus).

#### 3.2.1. First Advantage: Logistical Simplification (Convenience, Time Savings, and Transportation)

Telehealth was valued for its convenience. Participants were able to attend the appointment from their home, which made it comfortable for them to join the appointment in a familiar environment.

*I didn’t have to go to the doctor’s office so I really enjoyed that…I didn’t have to leave my house and the doctor was able to tell me though video call my results so it is just easier… Everything was fast and efficient without having to leave my house.*

*(P53)*

Telehealth required less time to get ready for the visit and provided more flexibility in scheduling since there was no need to travel.

*I don’t have to try to figure it out about, oh, let me make it before I pick up [granddaughter] from school. Oh, let me make it before the traffic starts. If it’s going up on I-8 [freeway], I’m not going to make it unless it’s between 10 and 1 o’clock… I don’t have to worry about my time and what time it is because I don’t have to worry about conditions on the road or my having to pick up my granddaughter.*

*(P14)*

Additionally, since commuting was not needed for the telehealth appointment, one participant noted how telehealth can help overcome transportation barriers. Transportation can be a challenge if participants do not have access to a car or need to rely on others for a ride, such as family members who may be busy working.

*The fact that they [appointments] can be at whichever time is convenient for me and not have to worry about who is going to take me and who is going to pick me up. It takes a weight off my shoulders when the appointments are in this method.*

*(P46)*

#### 3.2.2. Second Advantage: Financial Savings

Telehealth was noted as having the potential for monetary savings. Participants may avoid having to pay certain fees, such as a copay as a cost sharing charge under a health insurance plan, for a telehealth visit compared to the customary fee assessed for an in-person office visit.

*Saving on the copay...I just saved myself 20 bucks and got the same info… It makes me happy just knowing I didn’t have to spend money at all. Other people like going in. I’d rather do it on the phone for those same reasons, not having to spend money.*

*(P14)*

#### 3.2.3. Third Advantage: Faster Access and Longer Duration Spent with Clinicians

Another advantage reported with telehealth use is being able to have quicker access to clinicians, ranging from being offered an earlier appointment date to the time spent during the actual heath visit. Participants may have preferred a telehealth visit since it was clinician-initiated and there was no wait time to be seen by the clinician, as opposed to time spent waiting in the consultation room during an in-person visit.

*Well because you are basically being treated the moment you call them. Since my blood pressure was really high, I didn’t know what to do and my doctor was able to help me out immediately… I think it was a great experience.*

*(P2)*

Additionally, P54 noted that she felt that the telehealth visit felt less rushed with the clinician due to the potentially longer duration of the appointment.

*It [telehealth] has been good because I am able to tell my doctor about everything I feel, and the consultations are much longer on the phone than in-person. During an in-person consultation, they check my vitals, examine my lungs and heart. Sometimes the consultations take 5–7 min but when we are on the phone, the consultations usually last 15–20 min.*

*(P54)*

#### 3.2.4. Fourth Advantage: Presence of Family Members

Participants identified family support as having an important role in the participants’ healthcare experience. The physical presence of a family member was useful in helping communicate with the clinician, such as helping translate, asking additional questions on behalf of the patient, and providing clarification. Family members could continue to attend the appointments with the patients via telehealth, compared to limitations restricting others to accompany patients during an in-person visit.

*A family member can be there with me listening to what the doctor is saying. They might even ask questions that I would not be able to think of because either shocked or still trying to process the situation…The fact that I can have someone there helps clear my doubts and ask questions that I might not think about asking. The truth is there can be more than one person next to you and that gives you more heads to think. When I have them there, they are able to remember questions we had previously discussed that I would have completely forget to ask if I had been there by myself.*

*(P9)*

#### 3.2.5. Fifth Advantage: Safeguard from COVID-19 Exposure

Safety from potential COVID-19 exposure was another perceived benefit of telehealth. Participants were fearful of contracting COVID-19 at the clinic, particularly due to the risk of being around other people. During a telehealth appointment, exposure to COVID was minimized by avoiding a potential crowded and indoor space.

*But right now with COVID, I think video call is best… It is kinda scary when you go to the doctor sometimes because there may be a lot of people. Of course, they take precautions. Of the times that I have been to the radiology clinic, there have been a small amount of people. There might have been only four or five and I liked that there weren’t many people. Every seat was six feet apart and I liked that.*

*(P10)*

### 3.3. Disadvantages of Telehealth Use and Potential Suggestions to Address Disadvantages

Despite the perceived advantages of telehealth, the majority specified that they preferred in-person office visits over telehealth visits and, if given a choice, telehealth appointments via video rather than phone calls. There were three general themes identified as disadvantages of telehealth: (1) clinical care limitations (such as the clinicians’ inability to perform physical examinations), (2) diminished engagement with clinicians, (3) difficulty of interpreter accessibility, and (4) technological challenges (related to internet connectivity and audio issues). The main recommendations were aligned with the identified disadvantages of telehealth listed in Table 1, and both the identified disadvantages and suggested strategies to address them are discussed below.

#### 3.3.1. First Disadvantage: Clinical Limitations Due to Lack of Hands-On Care

Several participants had negative perceptions of telehealth related to the inability to have a physical examination performed. Some questioned the thoroughness of the health visit due to the lack of a hands-on clinical checkup, such as one participant who expressed some doubt.

*It is not the same looking at a camera and being able to physically be with them. On a call, the doctor won’t be able to see if your throat is swollen or if something is hurting. It does generate a form of insecurity… For example, let’s say I feel a lump, and we are talking on the phone. There is no way for the doctor to look it over because he is just on the phone.*

*(P1)*

Participants felt more assured if the clinician performed a hands-on physical rather than only relying on a verbal inquiry about the reported symptomology of the patient, particularly for appointments with an oncologist rather than a general practitioner.

*Also, being able to be with the doctor and have them check up on you. As much as I try not to worry, I sometimes worry about having something, an irregularity. So then the doctor can check my breasts and the glands on my throat, neck and underarms. After she checks and sees that there is nothing irregular, I can go home in peace. *

*(P46)*

##### Suggestion to Address the First Disadvantage: Consider the Nature of the Visit by Purpose and Severity of Medical Symptoms

A few participants referred to less urgent visits as “simple” or “general” appointments, where a discussion between the patient and clinician would suffice via telehealth. The types of discussion were based on various purposes that would not require a physical examination, such as an explanation about a new prescribed medication, a follow-up visit to touch base with the clinician, a discussion of test results, or an explanation of upcoming procedures.

*I think a video call should be used when you tell your doctor what is wrong with you and then they can decide if you actually do need to go to the doctor’s office or they could just give you a prescription over the video call. Kind of like a screening. *

*(P10)*

#### 3.3.2. Second Disadvantage: Limitations in Building Rapport and Engagement with Clinicians

Some participants perceived that there was less of a social connection with the clinician when conducted via telehealth, particularly in terms of communication and development of patient–physician rapport. P64 articulated that telehealth can restrict and limit patients’ sense of engagement.

*In-person it is much more personal because you get to properly communicate and look at each other face to face. I find it very important because I like to see their expressions and just have a face-to-face conversation. It is very important to trust your doctor and I feel like that can only be achieved by seeing each other in person.*

*(P6)*

In addition, telehealth visits were perceived to be less effective in fostering trust compared to an in-person office visit between patients and clinicians. P9 explained how face-to-face interaction provided better emotional support than a telehealth visit.

*It is not the same thing when they see you through video when you have cancer and not have them extend their hand. For them to tell you “To be honest, these results did not turn out how we expected.” That emotion is not the same one that they transmit through a phone as opposed to a face-to-face meeting. For example, if I get emotional and cry…You or a doctor might think “What do I do? How do I comfort her? I can’t give her a pat on the shoulder and tell her everything will be okay.”*

*(P9)*

##### Suggestion to Address the Second Disadvantage: Initial In-Person Visit and Follow-Up Telehealth Visits

A suggestion that can help foster the patient–clinician relationship relates to the timing of offering telehealth services in the continuum of care. The first appointment prior to introducing telehealth could be scheduled as an office visit so the patient is able to meet the clinician in-person, and then subsequent appointments could be scheduled as telehealth visits after establishing initial rapport.

*For my appointment with the doctor, I was asked if I wanted it to be an in-person consultation or on the phone. I picked in-person because I wanted to meet her and wanted the doctor to check how I was doing. The first time I met her we talked about my medications and everything in my file…I personally like to trust my doctors and I also want them to get to know me more.*

*(P2)*

#### 3.3.3. Third Disadvantage: Difficulties with Interpreter Assistance

Being able to have proper access to interpreters was also identified as a challenge in telehealth. Translation services may have been offered during the health visit, but the mechanism of delivery via telehealth encountered issues, such as the patient being unable to clearly hear the interpreter. P46 recalled an experience when the interpreter connected via a different platform and indirectly communicated with the clinician and patient who were on a separate platform.

*You know what happened, the problem was that the nurse had the translator on the phone. When one goes to the appointments in-person, there is a phone that they use to call in a translator. So during our video call, I was talking to the nurse through Zoom and she had the translator on speaker with one of those phones that they use. The translator never actually joined the meeting on Zoom. Because of that I was not able to hear what they were saying.*

*(P46)*

##### Suggestion to Address the Third Disadvantage: Joint Telehealth Visits with an Interpreter

One proposed strategy to overcome this barrier is to have an interpreter attend with the clinician on-site at the healthcare facility or to ask the interpreter to directly join the phone call or video chat for improved clarity.

*…having the translator actually attend the Zoom meeting would be helpful. Whether it be that they are in the same room with the doctor or nurse that I am talking to or if they could join the meeting using their own separate device.*

*(P46)*

#### 3.3.4. Fourth Disadvantage: Technology- and Logistics-Related Challenges

Technology-related issues were another disadvantage commonly reported by participants when using telehealth services since the use of video chat may be novel. For instance, digital literacy was low for nearly half of the participants who stated that they were unfamiliar with how to use the technology required for telehealth visits.

*I think I would struggle with the [video chat] link. I don’t know much about technology, computers, or smartphones. I barely started learning how to use my smartphone so I could see how it could be a bit challenging to open the link.*

*(P54)*

However, the issues that were encountered were not only attributed to technological advancements. Technical problems may arise, stemming from unstable internet connectivity and audio issues. Not being able to hear can hinder a productive visit if the patient cannot understand the clinician.

*This other time I was on a video call with my endocrinologist and translator, for some reason my audio sounded so odd. I could hear my audio cut off and sound like a robot so it wasn’t so easy to have a conversation… we just had to repeat everything over again and wait for the audio to sound better. It was important we waited until we could all hear clearly because I needed to ask questions about some medications that I was taking.*

*(P39)*

Participants also expressed the difficulties of having to remember information to log into a video chat. For instance, Zoom was a common video chat platform mentioned among participants for telehealth visits.

*To begin with, I don’t know how to make a video call… I used to have one email account but I forget the password to that one so I had to create a new one. So now I get mixed up between those two emails. When I am told to provide an email for the Zoom, I am not sure which one to give.*

*(P49)*

Telehealth visits also may encounter logistical challenges due to missed appointments. Missed appointments may translate into scheduling issues that may disrupt the continuity of cancer care.

*…I missed my session because I forgot [about telehealth appointment] and then she accidentally scheduled me for the date I had radiation so it has been like a month since I last saw her. I have to contact her department in order to fix that and have her schedule me for other days that don’t fall under my radiation days.*

*(P39)*

Another participant also commented about not being able to properly identify who was calling. She felt compelled to answer calls, even though she did not recognize the number, since she was waiting for the clinician to call.

*They’ll tell you sometimes that I have to answer every call that comes through until I get my call from [the clinic] because I don’t pick up numbers I don’t recognize, scam calls and all that stuff…So having to pick up scan scam calls is an inconvenience.*

*(P14)*

##### Suggestion to Address the Fourth Disadvantage: Tutorial and IT Assistance

Participants acknowledged that the use of such technology in telehealth services is a growing norm. It was suggested that if patients are taught about how to use video chats for telehealth visits, then they can learn to adapt and become more self-sufficient in accessing telehealth. A suggested strategy is for health systems to provide assistance to navigate the technology used for telehealth visits and improve patients’ digital literacy, such as offering a tutorial/training or one-on-one information technology (IT) assistance. In general, IT assistance may not be readily known or available for patients.

*For someone to show me how to use Zoom. I would like to take a course on that.* *We moved to these senior apartments not too long ago and since it’s only older people who live there, there is a classroom where they teach you how to use the computer. They are not doing it currently, but I think it would be good to take a class like that one day.*

*(P49)*

##### Additional Suggestion to Address the Fourth Disadvantage: Calls and Appointment Reminders

One suggestion to avoid missed telehealth appointments was to encourage a phone call reminder about an upcoming scheduled visit. For instance, P39 recommended a reminder to be sent prior to the scheduled time.

*I would like to have a reminder. I think it would be better if they called me like 15–20 min prior to my appointment so I could start filling out all those online forms and getting my medications ready…So that is definitely something that could really make it easier for me.*

*(P39)*

Another participant noted that if there was a narrow time frame set for the receipt of the telehealth appointment via telephone, then there would be likely less risk of picking up during a scam call.

*I haven’t encountered any problems other than I have to make sure that if I’m expecting a call, then it’s going to be between 1 and 2. I don’t have to deal with that [scam calls], then it’s like, OK, I got my appointment and now I’m not answering any other calls.*

*(P14)*

## 4. Discussion

The present study aimed to explore the perceived advantages and challenges/disadvantages of telehealth among Hispanic females diagnosed with breast cancer. The future of telehealth continues to grow and become more commonplace, as more patients may use and prefer this medium of care. The delivery of telehealth services in this study was discussed by participants via telephone (mobile or landline) and computers in general (no distinction between a tablet, laptop, or desktop). The major identified themes were in alignment with the previous literature, and potential strategies to mitigate the challenges were reported.

### 4.1. Advantages of Telehealth: Convenience, Time Savings, and Fiscal Savings

Our participants reported that a major advantage of telehealth was attributed to convenience and time savings, as similarly reported in prior research [12,14,15,17]. Participants noted that they were able to save time by avoiding traffic and not having to travel, which can be particularly beneficial for those who have a longer commute and must come from a rural area [12,14]. Additionally, participants mentioned the convenience of increased flexibility in scheduling and shorter wait times for telehealth compared to in-person visits, which is a sentiment that was echoed in other studies [14,15].

Financial benefits were another reported advantage, such as travel-related savings by conserving gas and avoiding parking costs [9,12]. Our study participants were of lower socioeconomic status. Although we did not measure the travel distance to the clinic, travel costs, including the price of gas, can impose a significant financial burden. Since January 2021, gas prices rose by USD 1 by the end of the year, with the highest costs for gasoline being charged on the West Coast [31]. In addition, study participants noted savings from avoiding a copay for telehealth visits. Waiving copays for a telehealth visit could provide financial relief, but this savings may only be temporary for some medical plans [32]. Any type of savings is important to consider since telehealth studies have shown that those who have lower socioeconomic status tend to use telehealth less [12,28].

Another major advantage of telehealth was the ability to have others present during the appointment. In general, family members and caregivers have commonly served as ad hoc interpreters for patients in healthcare settings [33,34]. However, during the COVID-19 pandemic, health centers imposed strict visitor policies, limiting in-person appointments mainly to patients [14]. Hence, telehealth was highly valued because it allowed family members to attend alongside and assist patients with translation and communication with clinicians (e.g., asking questions). It can also reinforce a core cultural value of familism for Hispanic patients, where collective reliance on the family may play a role in health decision-making [35].

### 4.2. Challenges to Telehealth and Suggestions

Similar to previous research [9,15,16,17], our study participants raised a concern about telehealth limiting hands-on care (e.g., physical examination). They may perceive their care as suboptimal due to the clinician’s inability to physically examine a lump or to check a physical pain via telehealth. Currently, there are no practice guidelines available or empirical data supporting virtual physical examinations for oncological care [17].

To address this clinical limitation, participants suggest scheduling in-person visits for appointments when a physical examination may be required or sensitive information needs to be conveyed. For instance, a poor cancer prognosis or the disclosure of a new cancer diagnosis by clinicians may be preferred during an in-person visit, and clinicians would be able to physically comfort patients, if needed [9,13,36]. Appointments via telehealth can be scheduled as a follow-up after an in-person visit or appointments to discuss medication management. It is important to consider the nature of the appointment to determine the appropriateness when deciding between an in-person or telehealth visit [12,15,16,37].

Our study participants also acknowledged the limitations of telehealth use in building rapport between patients and their clinicians. A systematic review with cancer patients [18] yielded similar results, stating that telehealth can be considered impersonal and lacking in personal engagement with clinicians. A proposed strategy is to consider the nature of the relationship with the clinician (e.g., first contact vs. established relationship) when scheduling a telehealth visit. An initial face-to-face appointment could be integral for establishing rapport when meeting a clinician for the first time, while subsequent appointments can be scheduled via telehealth for routine visits in order to maximize the benefits of both telehealth and in-person appointments. Trust can further foster in the patient–clinician relationship in telehealth visits after a relationship is first established in-person [38].

Another challenge of telehealth was the coordination of interpreter services. For example, one participant noted that the interpreter communicated with the clinician via phone while the clinician was meeting with the patient via video chat (e.g., Zoom) rather than all three being on the same video platform together. This can create confusion and negatively impact the quality of patient–provider communication. Reliance on the use of a formal interpreter is essential for healthcare equity. In response, participants suggested the capability of having the interpreter directly join a telehealth visit via video chat so multiple people are able to attend the appointment. In a systematic review comparing patient satisfaction between in-person, telephone, and video interpreting services, the majority preferred the use of a professional interpreter rather than an ad hoc interpreter (such family members). There was also no difference in satisfaction between the receipt of video and in-person interpreter services, where telephone interpreters were least preferred [39].

Lastly, digital literacy is a critical consideration during the onset of a rapid expansion of telehealth services. Consistent with the findings from previous studies [9,21,26,40], participants reported technology-related issues such as unfamiliarity with how to use video chat and a lack of stable internet connectivity. The digital divide in utilizing e-health technology can exacerbate disparities for certain populations, particularly racial and ethnic minorities, low-income communities, and older adults who may not have access or be familiar with such technology [21,23,40].

A potential strategy suggested by our participants is the introduction of a helpline that patients can call for IT assistance during business hours. This type of assistance could assist in overcoming the challenges of low digital literacy and help troubleshoot technological issues for telehealth visits. Our participants also addressed challenges in remembering how to log onto a video chat and forgetting about a telehealth appointment. A study by Gomez and colleagues [16] recommended patients receive a call 30 min prior to the telehealth appointment, which was similarly suggested in our study as a few minutes prior to the scheduled visit to help reduce missed appointments. Another potential reason for missed appointments is the inability to recognize the caller, which was also a concern reported in a 2022 study by Smith and colleagues [9]. Perhaps having a narrow time frame between the receipt of a reminder and a scheduled appointment could help prompt patients about the upcoming telehealth visit via telephone, particularly if the caller identification is not shown. Overall, our study participants’ suggestions can serve as potential recommendations to help overcome the challenges of telehealth and improve the patient healthcare experience.

### 4.3. Study Strengths and Limitations

This study contributes to the scientific literature by focusing on a sample that is normally not highly represented, including those with low English proficiency and minorities with breast cancer, and those underrepresented in research, such as participants of low socioeconomic status and Hispanic descent. However, it is noteworthy to mention some limitations in our study. One limitation is that our participants were recruited from one study site in an urban setting, and we used nonprobability sampling methods, which can limit the generalizability of findings to other Hispanic breast cancer patients living in different geographic locations (e.g., rural areas). Another limitation is that telehealth capacities may vary depending on the region, as rural hospitals were found to be less likely to have robust telehealth systems for patient engagement compared to those in metropolitan areas [41]. Future studies recruiting participants from multiple sites in both rural and urban areas may expand our understanding of potential barriers and needs for telehealth care. In addition, future studies can include family members to provide supplemental advantages and challenges/disadvantages of telehealth. Although family involvement in cancer care is common across race/ethnicity, the role of family is critical among Hispanic populations. Exploring families’ experiences and perspectives relating to patient care via telehealth may provide valuable information and guidance for telehealth practice improvement.

## 5. Conclusions

As we move into a different phase of the COVID pandemic as an endemic, this study provides insight and recommendations for improvements in telehealth use to better address the needs of patients with cancer. With the rapid onset of the pandemic, health systems had to quickly adjust and transform how care was provided, while cancer patients were faced with the double burden of having to overcome health challenges and racial and ethnic disparities due to telehealth challenges. It is imperative for clinicians to assess telehealth-related concerns among the underserved population and provide culturally appropriate services accordingly. Although the majority of study participants still preferred the traditional delivery of in-person care over telehealth services, healthcare providers should take into consideration the patient’s preference when possible and ensure equity in the delivery of care when maneuvering through the digital divide. More research is also needed to understand telehealth’s impact on the quality of cancer care. It is of equal importance to evaluate issues related to disparities due to the growing use of telehealth services, particularly for negatively affected groups, including the Hispanic community, those with low incomes, and people with limited English proficiency.

## Figures and Tables

**Table 1:** Participants’ sociodemographic characteristics and cancer-related information (N = 27).

Variable	N (%)/M (SD)
**Age (years)**	54.4 (11.6)
**Level of education**	
Less than high school	5 (18.5%)
High school/GED	12 (44.4%)
Some college	6 (22.2%)
College graduate	3 (11.1%)
Graduate degree	1 (3.7%)
**Annual household income**	
USD 30,000 or less	16 (59.3%)
USD 30,001 to USD 60,000	6 (22.2%)
Refused	5 (18.5%)
**Language of interview**	
Spanish	24 (88.9%)
English	3 (11.1%)
**Telecommunications access**	
Smartphone only	18 (66.7%)
Landline only	3 (11.1%)
Smartphone and landline	6 (22.2%)
**Year of breast cancer diagnosis**	
Less than a year	15 (55.6%)
Between 1 year and 2 years	7 (25.9%)
2 years to 3 years	2 (7.4%)
3 years to 4 years	1 (3.7%)
Over 4 years	2 (7.4%)
**Stage of breast cancer**	
Stage 0	1 (3.7%)
Stage 1	4 (14.8%)
Stage 2	11 (40.7%)
Stage 3	2 (7.4%)
Stage 4	3 (11.1%)
Unsure/do not know	6 (22.2%)

**Table 2:** Themes of telehealth use: advantages, disadvantages, and suggestions to address disadvantages.

Advantages of Telehealth	Disadvantages and Suggestions to Address Disadvantages of Telehealth
Simplification of logistical issues (convenience, time savings, and transportation) Financial savings Faster access to and longer duration with clinicians Presence of family members Safeguard from COVID-19 exposure	Clinical limitations due to lack of hands-on care Prioritization of appointment by the nature of the visit (purpose and severity) Limitations in building rapport and engagement with clinicians Initial visit conducted in-person, with follow-up visits via telehealth Difficulties with interpreter assistance Joint telehealth visits with an interpreter Technological and logistic-related challenges Tutorial of how to use video chat IT assistance provided Appointment reminders

## Data Availability

The datasets generated and analyzed during this study are available from the corresponding author on reasonable request.
